# A Novel Cell-Penetrating Peptide Derived from Human Eosinophil Cationic Protein

**DOI:** 10.1371/journal.pone.0057318

**Published:** 2013-03-04

**Authors:** Shun-lung Fang, Tan-chi Fan, Hua-Wen Fu, Chien-Jung Chen, Chi-Shin Hwang, Ta-Jen Hung, Lih-Yuan Lin, Margaret Dah-Tsyr Chang

**Affiliations:** 1 Institute of Molecular and Cellular Biology, National Tsing Hua University, Hsinchu, Taiwan, Republic of China; 2 Genomics Research Center, Academia Sinica, Taipei, Taiwan, Republic of China; 3 Department of Life Science, National Tsing Hua University, Hsinchu, Taiwan, Republic of China; 4 Department of Neurology, Taipei City Hospital, Zhongxiao Branch, Taipei, Taiwan, Republic of China; 5 Department of Medical Science, National Tsing Hua University, Hsinchu, Taiwan, Republic of China; University of Helsinki, Finland

## Abstract

Cell-penetrating peptides (CPPs) are short peptides which can carry various types of molecules into cells; however, although most CPPs rapidly penetrate cells *in vitro*, their *in vivo* tissue-targeting specificities are low. Herein, we describe cell-binding, internalization, and targeting characteristics of a newly identified 10-residue CPP, denoted ECP^32–41^, derived from the core heparin-binding motif of human eosinophil cationic protein (ECP). Besides traditional emphasis on positively charged residues, the presence of cysteine and tryptophan residues was demonstrated to be essential for internalization. ECP^32–41^ entered Beas-2B and wild-type CHO-K1 cells, but not CHO cells lacking of cell-surface glycosaminoglycans (GAGs), indicating that binding of ECP^32–41^ to cell-surface GAGs was required for internalization. When cells were cultured with GAGs or pre-treated with GAG-digesting enzymes, significant decreases in ECP^32–41^ internalization were observed, suggesting that cell-surface GAGs, especially heparan sulfate proteoglycans were necessary for ECP^32–41^ attachment and penetration. Furthermore, treatment with pharmacological agents identified two forms of energy-dependent endocytosis, lipid-raft endocytosis and macropinocytosis, as the major ECP^32–41^ internalization routes. ECP^32–41^ was demonstrated to transport various cargoes including fluorescent chemical, fluorescent protein, and peptidomimetic drug into cultured Beas-2B cells *in vitro*, and targeted broncho-epithelial and intestinal villi tissues *in vivo*. Hence this CPP has the potential to serve as a novel vehicle for intracellular delivery of biomolecules or medicines, especially for the treatment of pulmonary or gastrointestinal diseases.

## Introduction

Cell-penetrating peptides (CPPs) are peptides derived from proteins that can transport cargo such as nanoparticles, low molecular weight compounds, other peptides, proteins, and nucleic acids into cells [1]. CPPs may potentially be used during clinical procedures such as gene therapy and cancer treatment, and thus substantial efforts have been made to discover CPPs with suitable carrier properties [1], [2].

Most CPPs are rich in positively charged Arg and/or Lys residues, and are internalized after initially interacting with negatively charged cell surface glycosaminoglycans (GAGs), which cluster CPPs on outer membrane surfaces [3], [4]. Cell-surface GAGs are complex polysaccharides that participate in cell growth, differentiation, morphogenesis, migration, and bacterial/viral infections. Major vertebrate GAGs include heparan sulfate (HS), chondroitin sulfate (CS)/dermatan sulfate (DS), and hyaluronic acid (HA) [5], [6]. It has been shown that syndecan-4, a heparan sulfate proteoglycan (HSPG), accelerates the uptake of cationic CPPs penetratin and octa-arginine into K562 cells [7].

CPPs are usually divided into two groups [1], synthetic peptides such as oligoarginines which penetrate 293T cells [8], [9], and peptides derived from natural proteins such as TAT^47–57^ (GRKKRRQRRRP) from nuclear transcription activator Tat protein (TAT) of human immunodeficiency virus-1, which penetrates various cell types [10]. In the past two decades, 52 CPPs derived from natural proteins that can translocate across cell membranes have been reported [1], [11]. Twenty-eight of these CPPs including 15 viral protein-derived peptides, 7 animal modulator-derived peptides, 3 antimicrobial peptides, and 3 toxin-derived peptides have been demonstrated or predicted to interact with cell-surface HS before penetrating plasma membranes [1], [11], [12]. Most of these heparin-binding CPPs possess consensus heparin-binding motifs XBBXB or XBBBXXBX, where B is a basic amino acid and X is any amino acid. These peptides are further classified as cationic or amphipathic peptides [13]. Heparin-binding CPPs not only requires electrostatic interactions, but also uses aromatic residues for hydrophobic interactions with target cells [14]. However, little is known about how sequential aromatic and cationic residues affect the interactions of CPPs with cell-surface molecules.

Human eosinophil cationic protein (ECP) and eosinophil-derived neurotoxin (EDN) are secretory ribonucleases (RNases) released by activated eosinophils [15]. Both ECP (RNase 3) and EDN (RNase 2) possess antiviral and antiparasitic activities [15]. Interestingly, the RNase activity of ECP is much lower than that of EDN [16], although ECP has stronger antibacterial [15], [17] and cytotoxic activities [18]. In addition, ECP binds lipopolysaccharides and peptidoglycans tightly [19]. The *N*-terminal domain of ECP (residues 1–45) retains most of the antimicrobial properties [20]. Boix and colleagues identified residues 1–38 as responsible for the bactericidal activity [21] and found that a cavity created by residues A8–Q14, Y33–R36, Q40–L44, and H128–D130 could bind a HS disaccharide [22]. We have previously reported that cell-surface GAGs, especially HSPGs, act as receptors to promote ECP internalization *via* the macropinocytic pathway [23], resulting in apoptosis in Beas-2B cells [24]. The cytotoxicity of ECP was significantly reduced in mutant cell lines that lacked cell-surface HS or GAGs [23]. A sequential segment of ECP, ^34^RWRCK^38^, was subsequently identified as a core heparin-binding motif [25].

Very few CPPs derived from heparin-binding regions in proteins have been reported. Here two 10-residue peptides, ECP^32–41^ (RYRWRCKNQN) containing a novel heparin-binding motif of ECP, and EDN^32–41^ (NYQRRCKNQN) possessing a consensus heparin-binding motif in EDN [25], were synthesized and their cell-binding, GAG-binding, cell-penetrating, and cargo-transport activities were analysed. Interestingly, only ECP^32–41^ displayed CPP-like properties. The main endocytotic routes for ECP^32–41^ internalization were found to be temperature-sensitive and energy-dependent. ECP^32–41^ was able to deliver a small fluorescent molecule, a recombinant protein, and a peptidomimetic drug into cells. Moreover, an ECP^32–41^-tagged protein was preferentially routed to broncho-epithelial and intestinal villi tissues in rat. Here we demonstrate that ECP^32–41^ is the first heparin-binding CPP derived from a secretory human RNase, and we propose that it may serve as a new vehicle for intracellular cargo delivery and tissue targeting. It is a promising candidate for further molecular and cellular engineering investigations.

## Results

### ECP^32–41^ Internalization

**Figure 1:**
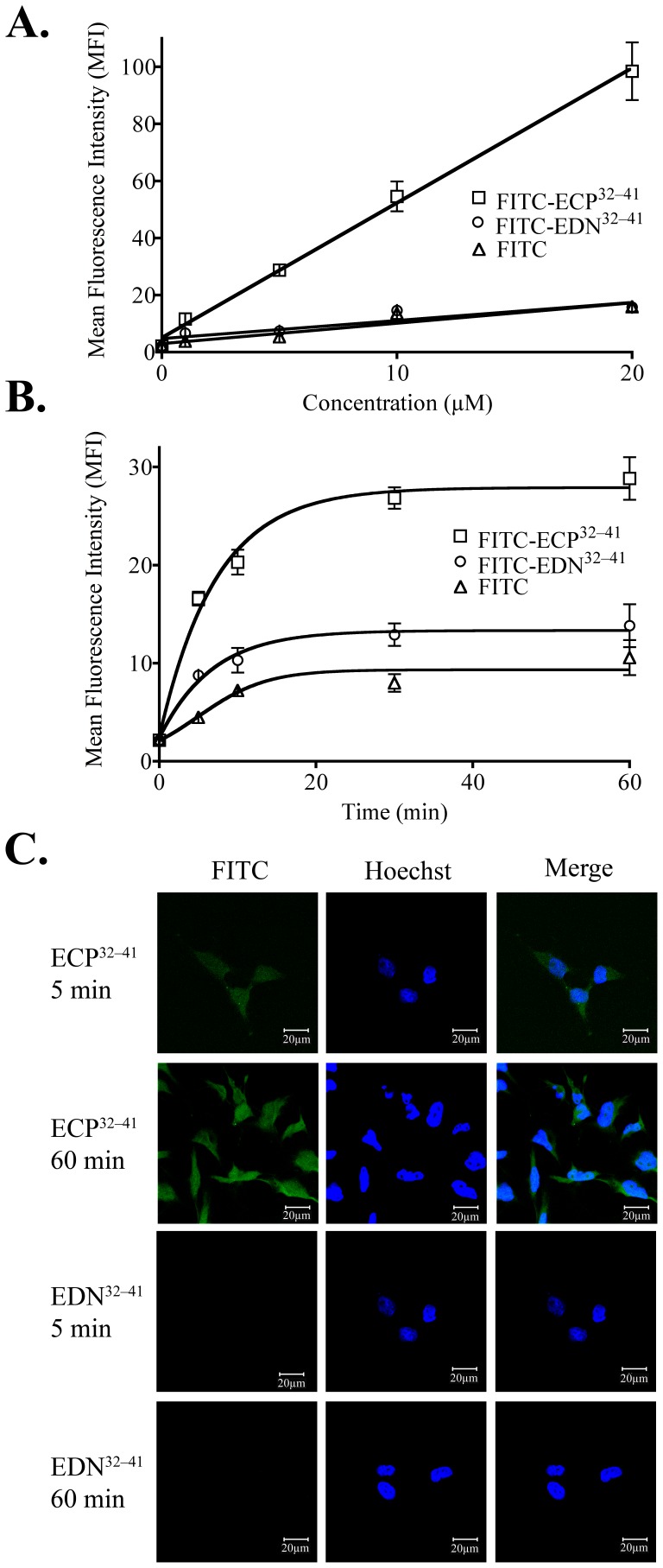
Internalization of ECP^32–41^ and EDN^32–41^. (A) Beas-2B cells were incubated with 1, 5, 10, or 20 µM FITC-ECP^32–41^, FITC-EDN^32–41^, or FITC at 37°C for 1 h. The cells were washed twice with 500 µl PBS, trypsinized at 37°C for 15 min, suspended in 500 µl PBS, and then subjected to flow cytometry. (B) Beas-2B cells were incubated with 5 µM FITC-ECP^32–41^, FITC-EDN^32–41^, or FITC at 37°C for 5, 10, 30, or 60 min. The cells were then treated as described in (A) and subjected to flow cytometry. The results in (A) and (B) are expressed as the mean ± standard deviation (S.D.), *n* = 3. (C) Beas-2B cells were incubated with 5 µM FITC-ECP^32–41^, FITC-EDN^32–41^, or FITC at 37°C for 5 or 60 min before CLSM. Nuclei were stained with Hoechst 34850. Scale bar: 20 µm.

Internalization of FITC-ECP^32–41^ and FITC-EDN^32–41^ was measured as the median ﬂuorescence intensity (MFI) of 6.0×10^5^ Beas-2B cells that had been treated with one of the FITC-labelled peptides (1 to 20 µM) at 37°C for 1 h, and then treated with trypsin to remove surface-bound peptides. FITC-ECP^32–41^ internalization was concentration dependent (Figure 1A), and at each concentration tested, the signal arising from FITC-EDN^32–41^ fluorescence was similar to that of the corresponding FITC control (Figure 1A). When Beas-2B cells were treated with 5 µM of a FITC-peptide at 37°C, the fluorescent signal for FITC-ECP^32–41^ increased within 5 min, and reached plateau at 30 min (Figure 1B). FITC-EDN^32–41^ penetrated the cells to a lesser extent during the 60 min incubation (Figure 1B). After addition of 5 µM ECP^32–41^, intercellular fluorescence was clearly detected 5 and 60 min later by CLSM, whereas a signal for intracellular EDN^32–41^ was not detected even after 1 h (Figure 1C). ECP^32–41^ therefore penetrated Beas-2B cells in a time- and concentration-dependent manner, whereas EDN^32–41^ did not act as a CPP, even though it contained a conventional heparin-binding motif.

### Influence of Sequence and Length on ECP^32–41^ Internalization

The sequences of ECP^32–41^ and EDN^32–41^ differ only at positions three and four, *i.e.*, Arg^3^ and Trp^4^ in ECP^32–41^ and Gln^3^ and Arg^4^ in EDN^32–41^ (Table 1). Because these two peptides internalized to significantly different extents (Figure 1), the importance of these residues was explored. Two ECP^32–41^ derivatives, ECP^32–41^R3Q and ECP^32–41^W4R, were synthesized, each with a single residue mutated to that found at the same position in EDN^32–41^, and were then tested for cell binding and internalization. Surprisingly, twice as much ECP^32–41^W4R bound to Beas-2B cells than did ECP^32–41^ and ECP^32–41^R3Q after 1 h incubation at 37°C (Table 2), indicating that an increase in the positive charge at position four led to stronger binding. Furthermore, when penetration of each peptide was assessed by FACS, only ECP^32–41^ internalized (Table 2). Replacement of an ECP^32–41^ residue with an EDN^32–41^ residue affected binding and internalization. The functionality of ECP^32–41^, as a CPP, could therefore be ascribed to both the W^3^R^4^ dipeptide sequence, and a nearly complete heparin-binding motif.

**Table 1:** Sequences and molecular weights of peptides.

Peptide	Sequence	Molecular weight (Da)	
ECP^32–41^	NYRWRCKNQN	1381	
EDN^32–41^	NYQRRCKNQN	1323	
ECP^32–41^R3Q	NYQWRCKNQN	1353	
ECP^32–41^W4R	NYRRRCKNQN	1351	
ECP^33–41^	YRWRCKNQN	1268	
ECP^32–40^	NYRWRCKNQ	1267	
ECP^33–40^	YRWRCKNQ	1153	
ECP^32–39^	NYRWRCKN	1139	
ECP^34–41^	RWRCKNQN	1104	
ECP^32–38^	NYRWRCK	1025	
TAT^47–57^	GRKKRRQRRRP	1493	
KLA	KLAKLAKKLAKLAK	1524	
KLA-TAT^47–57^	KLAKLAKKLAKLAKGRKKRRQRRRP	2999	
KLA-ECP^32–41^	KLAKLAKKLAKLAKNYRWRCKNQN	2887	

**Table 2:** Binding and penetrating activities of synthetic peptides in Beas-2B cells.

Peptide	Binding (%)^a^	Penetrating (%)^b^
ECP^32–41^	100	100
EDN^32–41^	19.4±0.05***	17.0±4.24***
ECP^32–41^R3Q	87.2±0.40	70.6±3.30*
ECP^32–41^W4R	549.9±1.00***	32.3±6.55**
ECP^33–41^	54.7±3.37*	48.2±2.48*
ECP^33–40^	72.4±4.68*	70.8±4.97*
ECP^34–41^	39.3±2.76**	34.3±2.41**
ECP^32–40^	90.7±7.79	82.0±9.42
ECP^32–39^	82.6±3.20	35.0±6.98**
ECP^32–38^	86.6±3.75	28.7±6.24**

X: amino-n-butyric acid. ND: not determined. The result is expressed as the mean ± S.D., *n* = 4.

*** *P*<0.001;

** *P*<0.01;

* *P*<0.05.

a Beas-2B cells were incubated with 5 *µ*M FITC-peptides at 4°C for 1 h, washed twice with PBS, and subjected to ELISA. The amount of FITC-ECP^32–41^ bound to Beas-2B cells was normalized to 100%.

b Beas-2B cells were incubated with 5 µM FITC-peptides at 37°C for 1 h. The cells were washed twice with 500 µl PBS, trypsinized at 37°C for 15 min, suspended in 500 µl PBS, and then subjected to flow cytometry. The fluorescence of cells treated with ECP^32–41^ was set as 100%.

To determine the optimal length (core composition) of cell penetrating properties, deletion of residues of ECP^32–41^ was individually carried out done from *N*-terminus and *C*-terminus. As expected, differences in the internalization were observed with the removal of amino acids from the *N*-terminus or *C*-terminus in the sequence (Table 2). *N*-terminally curtailed peptides, ECP^33–41^, ECP^34–41^ and ECP^34–40^ showed significantly 44%, 28% and 61% lower cell binding and thus decreasing internalization by 52%, 30% and 66% (Table 2). However, *C*-terminally curtailed mutants, ECP^32–39^ and ECP^32–38^ showed similar cellular binding as ECP^32–41^ but lower internalization than wild type ECP^32–41^ (Table 2). Most importantly, only ECP^32–41^ showed the highest cellular uptake, strongly suggesting that the length of our ECP-derived CPP was critical for internalization and residues from 32 to 41 were required.

### Effects of GAG on ECP^32–41^ Binding

**Figure 2:**
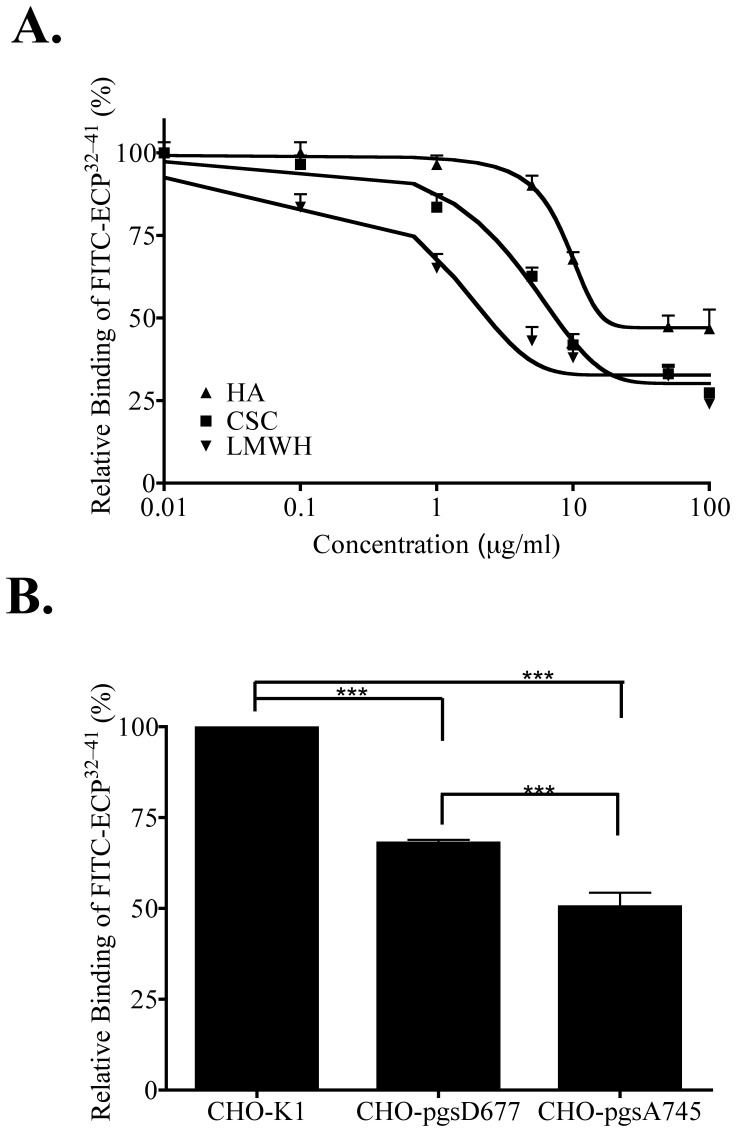
Cell-surface GAG-dependent binding of ECP^32–41^. (A) Beas-2B cells were treated with the indicated concentrations of LMWH, HA, or CS for 30 min prior to incubation with 5 µM FITC-ECP^32–41^ at 37°C for 1 h. After washed twice with PBS, cells were subjected to ELISA. The result is expressed as the mean ± S.D., *n* = 3. (B) CHO cells were incubated with 5 µM FITC-ECP^32–41^ at 4°C for 1 h, washed twice with PBS, and subjected to ELISA. The amount of FITC-ECP^32–41^ bound to CHO-K1 cells was normalized to 100%. The result is expressed as the mean ± S.D., *n* = 3. ***, *P*<0.001.

Cell-membrane GAGs including HS, CS/DS, and HA are necessary for CPP internalization [5], [6]. To assess the effect of GAGs on ECP^32–41^ cellular binding ability, soluble GAGs including LMWH, CSC, and HA were used as competitors to inhibit the attachment of ECP^32–41^ to Beas-2B cells. At concentrations between 0.01 and 1 µg/ml, LMWH, CSC, and HA inhibited ECP^32–41^ binding, with LMWH being the most effective and HA being the least. At concentrations exceeding 50 µg/ml, LMWH and CSC prevented approximately 70% of the normal ECP^32–41^ binding, whereas 53% inhibition was observed for 100 µg/ml HA (Figure 2A). Hence HS and CS might be involved in ECP^32–41^ binding to Beas-2B cells. To clarify the roles of cell-surface HS and CS, the binding of ECP^32–41^ to wild-type and two mutant strains of Chinese hamster ovary (CHO) cells was assessed by ELISA. CHO-pgsD677 cells do not express *N*-acetylglucosaminyltransferase and glucuronyltransferase, and therefore lack HS, but produce three times more CS than wild-type CHO-K1 cells [26]. CHO-pgsA745 cells are deficient in xylosyltransferase so that no GAG was present on the surface [27]. The amount of ECP^32–41^ bound to CHO-pgsA745 cells was found to be 52% less than that bound to CHO-K1 cells, suggesting that GAG was required for binding (Figure 2B). Additionally, a 31% reduction in ECP^32–41^ binding was observed for CHO-pgsD677 cells, even though the cells expressed much more CS than CHO-K1 cells (Figure 2B). GAGs, and especially HSPGs, are therefore crucial for the initial interaction of ECP^32–41^ with cell surfaces.

### Effect of HS on ECP^32–41^ Internalization

**Figure 3:**
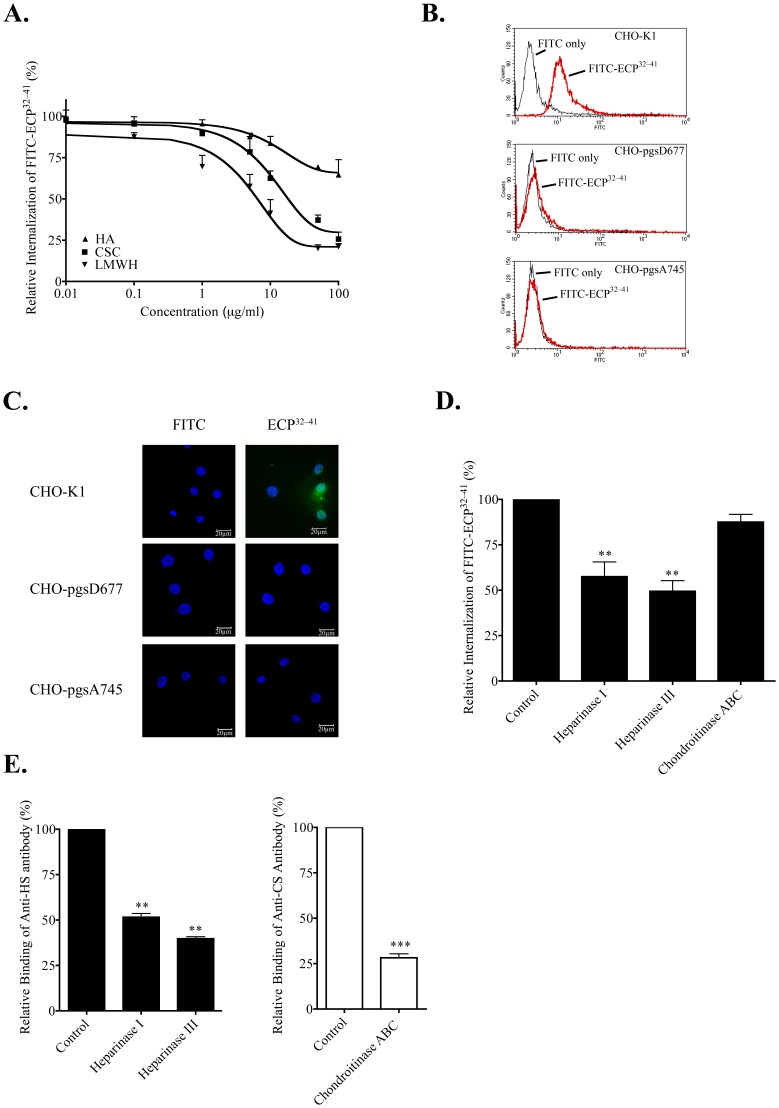
HS-dependent ECP^32–-41^ internalization. (A) Beas-2B cells were treated with the indicated concentrations of LMWH, CS, or HA for 30 min prior to incubation with 5 µM FITC-ECP^32–41^ at 37°C for 1 h. The cells were washed twice with 500 µl PBS, trypsinized at 37°C for 15 min, suspended in 500 µl PBS, and subjected to flow cytometry. The result is expressed as the mean ± S.D., *n* = 3. (B) Samples of wild-type and mutant CHO cells were each incubated with 5 µM FITC-ECP^32–41^ at 37°C for 1 h and then subjected to flow cytometry. (C) Samples of wild-type and mutant CHO cells were incubated with 5 µM FITC-ECP^32–41^ at 37°C for 1 h., then washed twice with 1 ml PBS, and fixed for CLSM. Nuclei were stained with Hoechst 34850. Scale bar: 20 µm. (D) Beas-2B cells were treated with heparinase I, heparinase III, or chondroitinase ABC for 2 h prior to incubation with 5 µM FITC-ECP^32–41^ at 37°C for 1 h. The cells were washed twice with 500 µl PBS, trypsinized at 37°C for 15 min, suspended in 500 µl PBS, and subjected to flow cytometry. Untreated cells served as the controls. The fluorescence of cells treated with FITC-ECP^32–41^ was set to 100%. The result is expressed as the mean ± S.D., *n* = 3. **, *P*<0.01. (E) Beas-2B cells were treated with heparinase I, heparinase III, or chondroitinase ABC for 2 h. After stained with anti-HS or anti-CS monoclonal antibodies, washed twice with 500 µl PBS, and hybridized with FITC-conjugated anti-mouse secondary antibody, cells were suspended in 500 µl PBS and subjected to flow cytometry. Untreated cells served as the control. The fluorescence of the untreated cells was set to 100%. The result is expressed as the mean ± S.D., *n* = 3. **, *P*<0.01 and ***, *P*<0.001.

To further investigate the involvement of GAG in ECP^32–41^ internalization, Beas-2B cells were incubated with LMWH, CS, and HA prior to treatment with ECP^32–41^. The resulting inhibition profiles (Figure 3A) were similar to those for binding Beas-2B cells (Figure 2A), and the effectiveness of LMWH, CS, and HA as inhibitors decreased in the same order. LMWH and CS (each at 10 µg/ml) decreased ECP^32–41^ internalization by 58% and 38%, respectively. HA treatment was less effective however, and only a 35% decrease was observed at high concentration of 100 µg/ml. Both HS and CS appear to facilitate ECP^32–41^ binding and internalization.

A significant fluorescence shift reflecting FITC-ECP^32–41^ internalization was observed for CHO-K1 cells but not for CHO pgsD-677 or CHO pgsA-745 cells (Figure 3B). ECP^32–41^ internalization was also clearly observed for CHO-K1 cells but not for CHO pgsD677 or pgsA745 cells, when monitored by CLSM (Figure 3C). HS, instead of CS, is therefore the major ECP^32–41^ receptor.

To further confirm that cell-surface HS, rather than CS, was involved in ECP^32–41^ internalization, Beas-2B cells were treated with heparinase I, heparinase III or chondroitinase ABC for 2 h, and then with FITC-ECP^32–41^ for 1 h prior to measuring the cell fluorescence signal (Figure 3D). Pre-treatment of Beas-2B cells with heparinase I or heparinase III decreased ECP^32–41^ internalization by 43% and 51%, respectively. CS depletion had little effect on ECP^32–41^ internalization, thus heparinase, but not chondroitinase ABC, inhibits ECP^32–41^ internalization.

To verify that selective cell-surface polysaccharides had been enzymatically removed, the treated cells were subsequently incubated with anti-HS or anti-CS monoclonal antibodies. As expected, heparinase I and heparinase III had decreased the amount of HS on Beas-2B cell surfaces by 48% and 61%, respectively (Figure 3E, left panel), and chondroitinase ABC removed 72% of the cell-surface CS (Figure 3E, right panel). The HS that remained on Beas-2B cell surface accounted for the observed ECP^32–41^ internalization into heparinase-treated cells. Conversely, even though 70% of the initial cell-surface CS had been removed, ECP^32–41^ internalization was hardly affected–thus as concluded above, HS, rather than CS, is responsible for ECP^32–41^ internalization.

### Temperature and Energy Dependences of ECP^32–41^ Internalization

**Figure 4:**
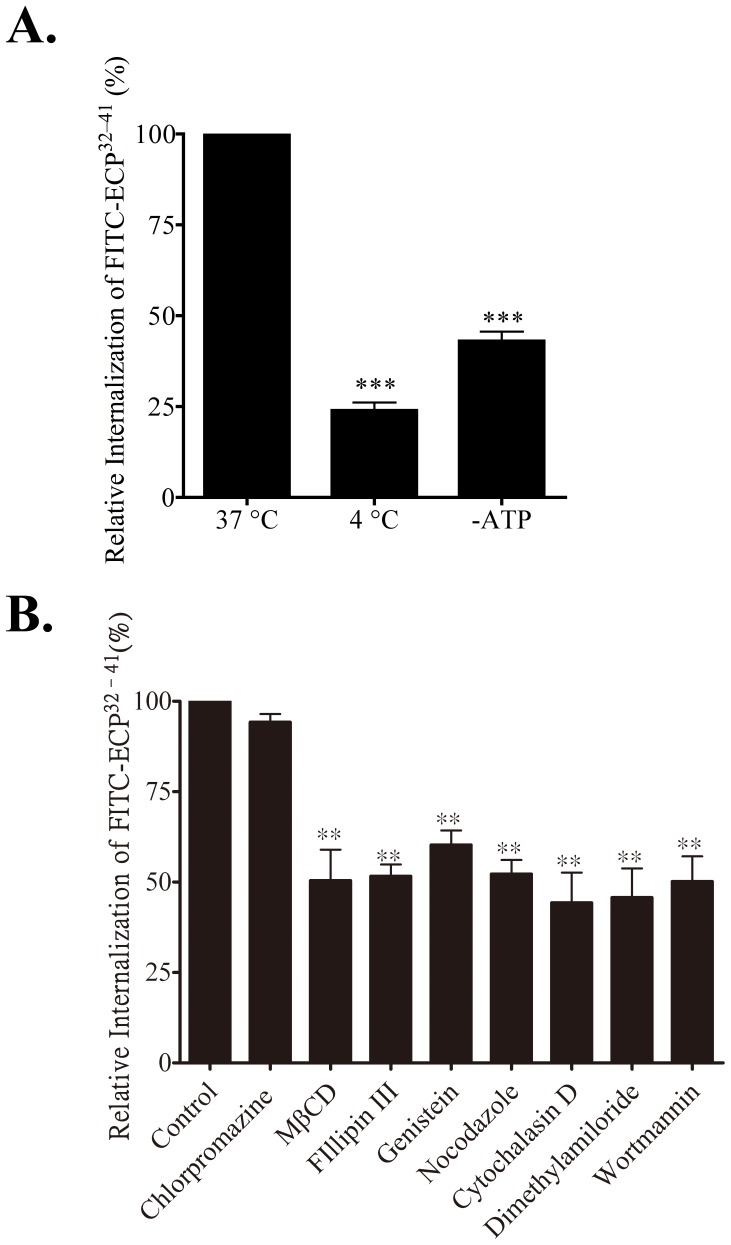
Internalization pathway of ECP^32–41^. (A) Beas-2B cells were incubated at 37°C, 4°C, or with ATP depletion at 37°C for 30 min and then incubated with of 5 µM FITC-ECP^32–41^ for 1 h. The cells were washed twice with 500 µl PBS, trypsinized at 37°C for 15 min, suspended in 500 µl PBS, and subjected to flow cytometry. The fluorescence of cells treated with ECP^32–41^ was set to 100%. The result is expressed as the mean ± S.D., *n* = 3. ***, *P*<0.001; **, *P*<0.01. (B) Beas-2B cells were incubated with the indicated endocytic inhibitors at 37°C for 30 min, followed by addition of 5 µM FITC-ECP^32–41^ at 37°C for 1 h. Cells were then treated as described in (A). The result is expressed as means ± S.D., *n* = 3. **, *P*<0.01.

CPPs enter cells by two routes: direct translocation through lipid bilayers or energy-dependent vesicular mechanisms referred to as endocytosis [28]. Direct CPP translocation is usually observed when the CPP concentration is above 10 µM [28]. To characterize the mechanism(s) of ECP^32–41^ internalization at low concentrations (≤5 µM), we investigated the effect of cellular ATP depletion and low incubation temperature–both of which were expected to inhibit endocytosis. FITC-ECP^32–41^ internalization was inhibited by 76% at 4°C, compared to 37°C (Figure 4A), when cell samples were first incubated at these temperatures for 30 min prior to addition of 5 µM FITC-ECP^32–41^. Pre-incubation with sodium azide and deoxyglucose, which depleted the cellular ATP pool, inhibited FITC-ECP^32–41^ internalization by 57%. ECP^32–41^ internalization is therefore, temperature- and energy-dependent, indicating that, at low concentrations of ECP^32–41^, the main internalization route is endocytic in nature.

### ECP^32–41^ Internalization *via* Lipid-raft Dependent Macropinocytosis

Endocytic pathways are generally grouped into four categories: clathrin- and caveolin-mediated pathways, macropinocytosis, and other less-well characterized clathrin- and caveolin-independent mechanisms [29]. Some of these pathways are also lipid-raft dependent [29]. We pretreated Beas-2B cells with endocytic inhibitors to identify the pathways involved in ECP^32–41^ internalization. Chlorpromazine, an inhibitor of clathrin-mediated endocytosis, did not affect FITC-ECP^32–41^ internalization (Figure 4B), suggesting that clathrin-mediated endocytosis was not involved. The lipid-raft pathway inhibitors methyl-β-cyclodextrin and genistein inhibited FITC-ECP^32–41^ internalization by 48% and 40%, respectively. Cellular uptake of ECP^32–41^ reduced 50% in the presence of filipin III which depleted lipid raft on cell membrane, also suggesting that lipid raft-dependent endocytosis was involved in ECP^32–41^ internalization. Nocodazole and cytochalasin D, which blocked cytoskeleton polymerization and consequently phagosome and macropinosome formation, respectively, reduced FITC-ECP^32–41^ internalization by 48% and 56%, respectively. Dimethyl amilorides, an inhibitor of the Na^+^/H^+^ ion exchange pump resulting in the cessation of macropinocytosis, and wortmannin, an inhibitor of both macropinocytosis and clathrin-mediated endocytosis, inhibited internalization by 50% and 53%, which indicated that macropinocytosis was involved. Lipid rafts are therefore involved in ECP^32–41^ internalization, and two pathways appear to govern ECP^32–41^ internalization: actin-dependent endocytosis and lipid-raft macropinocytosis.

### Cytotoxic Effects of ECP^32–41^

**Figure 5:**
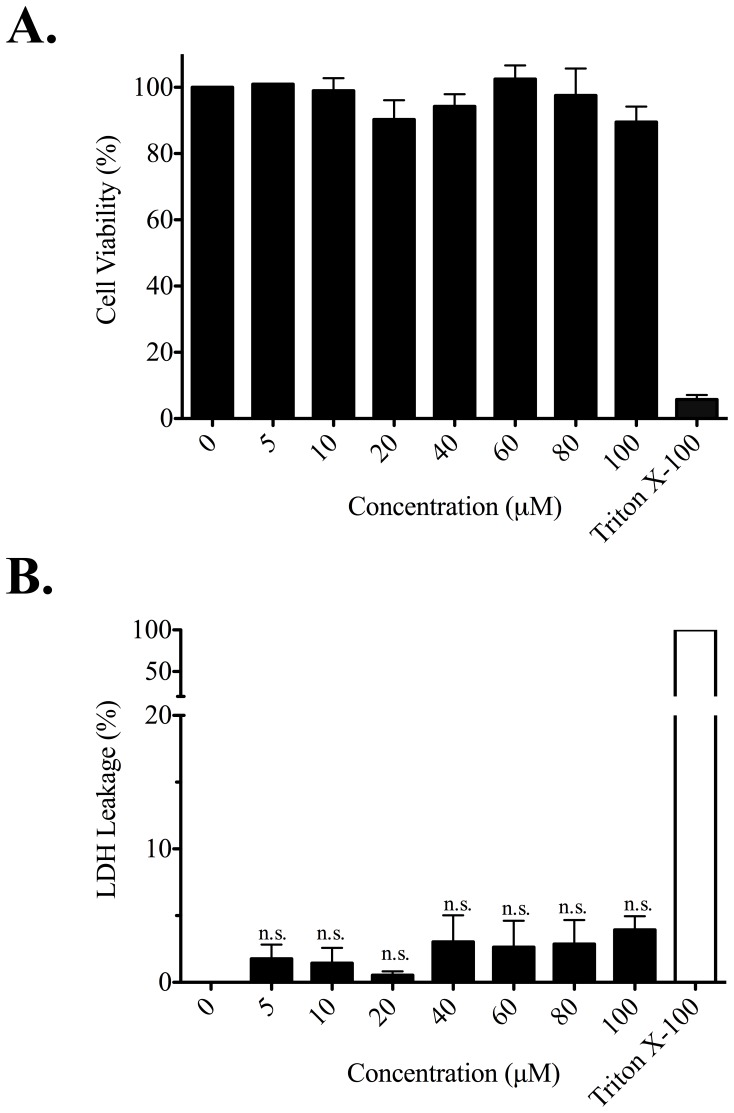
Cytotoxicity and membrane disruption by ECP^32–41^. Beas-2B cells were grown in serum-free medium in the presence of ECP^32–41^ at indicated concentrations for 24 h. (A) The cytotoxic effect of ECP^32–41^ was measured by MTT assay. The cell viability of untreated cells was set to 100%. Cells treated with 0.1% Triton X-100 was used as a positive control. (B) The membrane disruption by ECP^32–41^ was measured by LDH assay. LDH released from cells lysed with 0.1% Triton X-100 in medium was defined as 100% leakage and LDH released from untreated cells was set as 0% leakage. The result is expressed as means ± S.D., *n* = 3. no significance (n.s.).

To get a comprehensive analysis of toxic profiles induced by ECP^32–41^, cytotoxic and membrane disruptive properties of ECP^32–41^ were analysed by 3-(4,5-dimethylthiazol-2-yl)-2,5-diphenyltetrazolium (MTT) and lactate dehydrogenase (LDH) leakage assay, respectively. Beas-2B was treated with ECP^32–41^ up to 100 µM at 37°C for 24 h. No sign of any negative effects in cell viability were observed after treatment with ECP^32–41^ (Figure 5A) and no significant changes (*P*>0.05) in LDH levels were found between ECP^32–41^ treated and untreated cells (Figure 5B). These results demonstrated that treatment of cells with ECP^32–41^ had no effects on cytotoxicity and membrane disruption.

### *In vitro* Delivery of Proteins and Peptides by ECP^32–41^ into Cells

**Figure 6:**
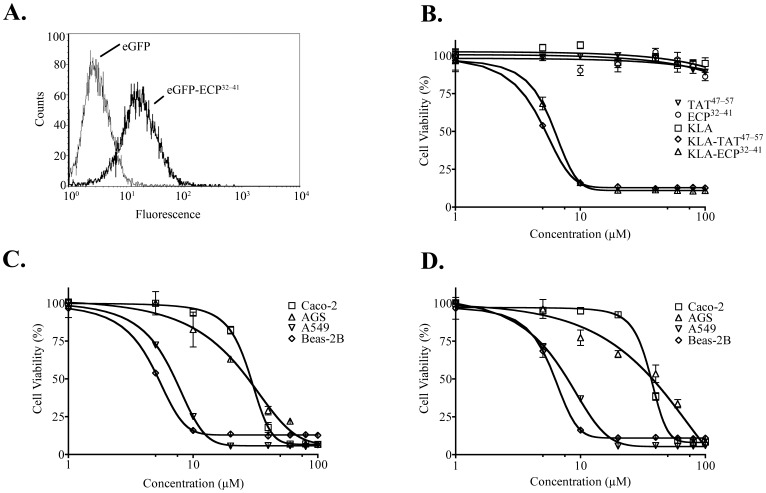
*In vitro* delivery of a protein and a peptide by ECP^32–41^. Beas-2B cells were incubated with 10 *µ*M eGFP or eGFP-ECP^32–41^ at 37°C for 1 h. The cells were then washed with 500 µl PBS, trypsinized at 37°C for 15 min, suspended in 500 µl PBS, and subjected to flow cytometry. (B) Beas-2B cells were treated with the indicated concentrations of TAT^47–57^, ECP^32–41^, KLA, KLA-TAT^47–57^ or KLA-ECP^32–41^ at 37°C for 24 h, and their cytotoxic effects were determined by the MTT assay. Cytotoxic effects of (C) KLA-TAT^47–57^ and (D) KLA-ECP^32–41^, at the indicated concentrations, on Beas-2B, A549, Caco-2, and AGS cells were assessed by the MTT assay after incubation at 37°C for 24 h. The cell viability untreated cells was set to 100%. The result is expressed as the mean ± S.D., *n* = 3.

The ability to mediate cellular uptake of normally impermeable small molecules, proteins, and peptides is an important functional characteristic of CPPs [28]. To determine what type(s) of cargo ECP^32–41^ could deliver, first, eGFP (28 kDa) was fused to ECP^32–41^ so that internalization of ECP^32–41^ could be monitored by flow cytometry. A fluorescent signal shift was clearly observed after incubating Beas-2B cells with eGFP-ECP^32–41^, indicating that ECP^32–41^ successfully delivered eGFP into the cells (Figure 6A).

To determine ECP^32–41^ penetration into the cell in terms of time and intracellular localization, the cytosolic and endosomal fractions of Beas-2B cells were isolated by subcellular fractionation after treatment with eGFP-ECP^32–41^. Beas-2B cells were incubated with eGFP or eGFP-ECP^32–41^ (20 µM) at 4°C for 1 h and then shifted to 37°C for further incubation for 1 h, 2 h, 3 h and 4 h separately. Cells were homogenized and fractionated by floatation in Percoll gradients separating cytoplasmic and endosomal fractions. Neither eGFP-ECP^32–41^ nor eGFP signal was detected along with Actin in cytoplasm even after 4 h incubation (Figure S1A). In terms of endosomal fraction, eGFP-ECP^32–41^ signal was detected along with LAMP-1 in endosomal fraction after 1 h incubation, the accumulated amount reached maximum at 2 h and then gradually decreased (Figure S1B). In contrast, eGFP signal was not detected even after 4 h treatment. These results suggested that larger cargo (eGFP-ECP^32–41^) remained in endocytic vesicles for at least 4 h.

In general, CPPs should not be cytotoxic when serving as viable delivery vehicles, the effect of ECP^32–41^ on cell viability was assayed using MTT assay with a well characterized CPP, TAT^47–57^, as a control [10]. At 100 µM, neither peptide affected cell viability (Figure 5A, Figure 6B); thus ECP^32–41^, unlike full-length ECP [23], [24], could potentially serve as a delivery vehicle. To assess the ability of ECP^32–41^ to deliver a small cargo, a peptidomimetic drug, KLA, which contained a proapoptotic domain that induces mitochondrial swelling but did not affect the plasma membrane, was chosen as the cargo [30], [31]. The *in vitro* internalization of KLA conjugated to TAT^47–57^ and ECP^32–41^ was evaluated by monitoring cell viability. As expected, KLA alone was not cytotoxic, whereas KLA-TAT^47–57^ and KLA-ECP^32–41^ had similar cytotoxic effects on Beas-2B cells when incubated at 37°C for 24 h (Figure 6B). KLA-TAT^47–57^ and KLA-ECP^32–41^ induced cell death in a concentration-dependent manner with half maximal effective concentrations (EC_50_) of 5.64 µM and 6.08 µM, respectively. ECP^32–41^ may therefore be useful as a delivery vehicle for functional peptides.

To investigate whether ECP^32–41^ maintains its penetration property after coupling with cargos, KLA-ECP^32–41^ was mixed with indicated concentrations of LMWH, CSC or HA prior to incubation with Beas-2B cells, followed by MTT assay. In the presence of 25 µg/ml of LMWH or CSC, the cytotoxicity of KLA-ECP^32–41^ was significantly reduced (Figure S2), inferring that KLA-ECP^32–41^ penetration decreased due to competition with LMWH or CSC. However, HA had no inhibitory effect on cytotoxicity of KLA-ECP^32–41^, similar to GAG influence on FITC-ECP^32–41^ internalization (Figure 3A). These results suggested that intracellular delivery of cargo-ECP^32–41^ relied on cell surface HS, resembling the case of ECP^32–41^ peptide alone.

To further explore which cell types ECP^32–41^ could enter, the cytotoxic effects of KLA-TAT^47–57^ (Figure 6C) and KLA-ECP^32–41^ (Figure 6D) on the human cell lines, lung A549, and digestive-tract Caco-2 and AGS cell lines were examined. The EC_50_ values for KLA-TAT^47–57^ and KLA-ECP^32–41^ in Beas-2B, A549, Caco-2 and AGS cells were summarized in Table 3. For the lung cell lines, the EC_50_ values of KLA-ECP^32–41^ and KLA-TAT^47–57^ on Beas-2B and A549 cells were quite similar (5 to 7 µM. However, for the digestive-track cell lines, EC_50_ values of KLA-ECP^32–41^ were respectively 1.6- and 2.42- fold higher than those of KLA-TAT^47–57^ in Caco-2 and AGS cells, presumably due to higher expression of HSPGs on lung cells or higher KLA resistance of gastrointestinal cell lines.

**Table 3:** Half maximal effective concentration of KLA-TAT^49–57^ and KLA-ECP^32–41^.

Cell line	EC_50_ (µM)	
	KLA-TAT^47–57^	KLA-ECP^32–41^
Beas-2B	5.64±0.37	6.08±0.26
A549	6.84±0.13	7.17±0.39
Caco-2	21.79±0.63	35.07±0.77
AGS	24.67±5.54	59.75±6.82

### Tissue Targeting of ECP^32–41^ in an Animal Model

**Figure 7:**
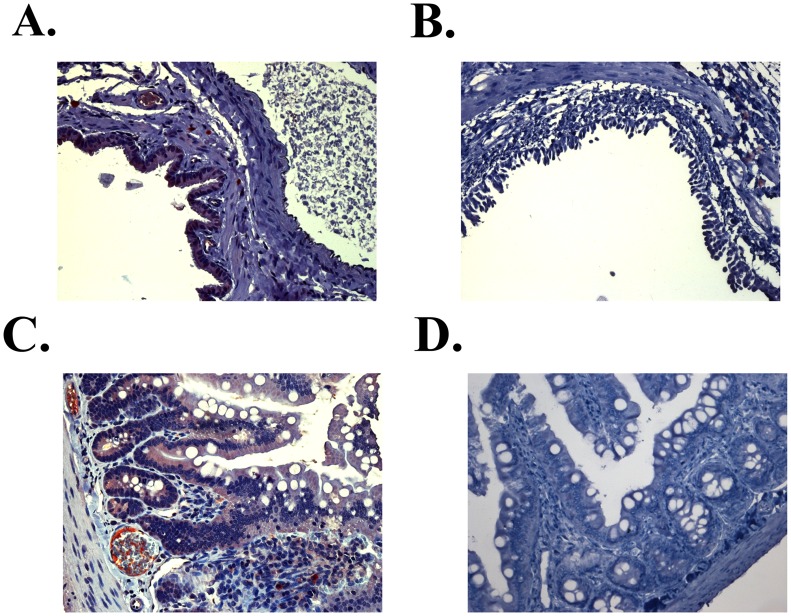
Tissue-specific localization of ECP^32–41^. Immunohistochemical staining of ECP^32–41^ was detected by Supersensitive Non-Biotin HRP Detection System. Representative images of eGFP-ECP^32–41^ (red) in (A) broncho-epithelial and (C) and the epithelium of intestinal villi 1 h after intravenous injection of eGFP-ECP^32–41^ were shown. Signals of eGFP were not detected in (B) broncho-epithelial and (D) intestinal villi tissue sections 1 h after intravenous injection of eGFP. (Magnification in all panels, 200×).

GAG expression is related to cell differentiation and growth [32], and specific HPSGs are differentially expressed in different cell types [33]. To delineate tissue targeting by ECP^32–41^ *in vivo* and to develop potential applications, eGFP-ECP^32–41^ and eGFP were separately injected into the circulatory system of specific-pathogen-free rats through tail veins. The tissues were immunohistochemically stained with anti-eGFP antibody. Interestingly, 1 h after injection, significant eGFP-ECP^32–41^ signals were detected in broncho-epithelial and intestinal villi tissues (Figure 7A, 7C), which was quite similar to tissue distribution as ECP [23]. eGFP alone was not detected in these tissues (Figure 7B, 7D). As is known that mammalian mucosal cells are rich in HSPGs, ECP^32–41^ may potentially be used for *in vivo* targeting of broncho-epithelial and intestinal villi tissues.

## Discussion

CPPs are a class of peptides differing in sequence, size, and charge that can translocate across plasma membranes. In this study, a newly identified CPP corresponding to residues 32–41 of human ECP (ECP^32–41^) was characterized. ECP^32–41^ delivered a small, fluorescent compound (Figure 1), a recombinant protein (Figure 6A), and a peptidomimetic drug (Figure 6D) into Beas-2B cells, and targeted specific rat tissues *in vivo* (Figure 7), showing that it can act as a delivery vehicle in both types of environments.

ECP is a multifunctional protein with ribonucleolytic, cytotoxic, membrane-disrupting, antibacterial, antiparasitic, antiviral, heparin-binding, and cell-penetrating activities. Boix and colleagues identified the heparin-binding residues in ECP as A8–Q14, Y33–R36, Q40–L44, and H128–D130 [22], and we have previously shown that residues 34–38 comprise a critical heparin-binding sequence, and substitution of residues in the ECP sequence ^34^RWRCK^38^ with alanine resulted in decreased cell-penetrating activity [25]. Here ECP^32–41^ is identified as the first CPP derived from a human RNase sequence.

Previous reports have suggested that the guanidinium group of arginine rather than lysine or histidine side-chains is necessary for CPP activity [34], [35]. Addition of tryptophan to heptaarginine peptide increases its uptake efficiency [36]. And further, cellular internalization of tryptophan distributed along (RWRRWRRWRRWR) shows higher efficiency than heptaarginine with tryptophan at the *N*-terminus [37]. Interestingly, although both ECP^32–41^ and EDN^32–41^ possess heparin-binding sequences differing only at two positions, they have very dissimilar cell-binding and internalization activities. ECP^32–41^R3Q and ECP^32–41^ bound Beas-2B cells similarly (Table 2), but ECP^32–41^R3Q did not penetrate cells as ECP^32–41^ did (Table 2). Additionally, although ECP^32–41^W4R had the strongest affinity for Beas-2B cell among those peptides tested (Table 2), it did not penetrate the cells (Table 2), possibly due to its tight binding to cell-surface GAGs [38]. Residues R^3^ and W^4^ in ECP^32–41^ thus appeared to be crucial for internalization. The two arginines adjacent to W^4^ in ECP^32–41^ possibly interacted with negatively charged cell-surface HSPGs, thereby promoting binding. Taken together, the positively charged arginines and the aromatic tryptophan are necessary for ECP^32–41^ internalization.

Most viral-derived CPPs are rich in basic amino acids [1]. For example, flock house virus coat peptide (residues 35–49, RRRRNRTRRNRRRVR) is extensively used as a CPP, it can interact with sulfated proteoglycans and negatively charged cell-membrane phospholipids [39]. Interestingly, internalization of the amphipathic peptide penetratin (RQIKIWFQNRRMKWKK) requires electrostatic interactions between basic residues and HS and the presence of aromatic residues, especially tryptophan, for insertion into a lipid bilayer [40]. Although the length of ECP^32–41^ is comparable to that of TAT^47–57^ and the antimicrobial CPP SynB3 (RRLSYSRRRF), physical characteristics of these three CPPs differ. TAT^47–57^ and SynB3 both have *p*I values of 12, owing to the presence of many cationic residues, whereas ECP^32–41^ has only two arginines and one lysine with a *p*I value of 10.05. Interestingly, the percentage of basic residues (50%) in CyLoP-1 (CRWRWKCCKK) from crotamine [41], one of the main toxins in rattlesnake venom, is similar to that of ECP^32–41^ (40%). A consensus motif RWRXK (where X is any amino acid) is present in both CyLoP-1 [41] and ECP^32–41^. Likewise, internalization of both CyLoP-1 and ECP^32–41^ requires positively charged residues and non-polar residues, especially tryptophan [41]. Moreover, tryptophan residues are preferentially oriented parallel to the membrane and required for membrane penetration of CPP [42]. ECP^32–41^, located in a flexible loop structure in intact ECP [22], showed random coil property as determined by circular dichroism spectroscopy (data not shown). Flexibility of structure has been suggested as a favorable property for direct membrane penetration for CPPs, as it might allow efficient cell entry [34], [40]. In summary, the presence of aromatic W^4^, the binding by cationic R^3^ and R^5^ to cell surface GAGs, and flexible backbone structure probably all contribute to ECP^32–41^ internalization.

Three lines of direct evidence indicated that cell-surface HS was involved in ECP^32–41^ endocytosis. First, soluble HS significantly decreased FITC-ECP^32–41^ internalization into Beas-2B cells (Figures 2A, 3A). Second, cell-surface HSPGs facilitated FITC-ECP^32–41^ binding because wild-type CHO cells uptook more FITC-ECP^32–41^ than did HS- and GAG-deficient CHO cells (Figures 2B, 3B). Third, removal of cell surface HS reduced FITC-ECP^32–41^ internalization into Beas-2B cells (Figures 3C, 3D). Therefore, cell surface HSPGs mediate ECP^32–41^ internalization.

In general, three steps are involved in CPP internalization: CPPs first bind to cell surface GAGs, then they move through the cell membrane, and finally they are released into the cytoplasm [43]. Initially, CPPs were thought to bind and directly cross plasma membranes *via* a receptor- and energy-independent path [44], [45]. However, internalization mechanisms, in addition to direct translocation, *e.g.*, clathrin-mediated, caveolin-mediated, macropinocytotic, and clathrin- and caveolin-independent endocytosis, have been reported [29]. Moreover, most CPPs employ two or more internalization pathways [44]. We have previously demonstrated that ECP internalization into Beas-2B cells occurs *via* HS-facilitated and lipid raft-dependent macropinocytic routes [23]. We have now found that HSPGs act as receptors or attachment factors for ECP^32–41^ internalization (Figure 3). Additionally, ECP^32–41^ internalized at 4°C, albeit with a lower efficiency than at 37°C, and endocytotic inhibitor screening suggested that lipid raft-dependent macropinocytic routes were also involved (Figure 4B). The internalization routes of ECP and ECP^32–41^ therefore, appear to be similar [23]. However, nocodazole blocked internalization of only ECP^32–41^ (Figure 4B) but not that of ECP [23], thus multi-endocytic routes should be involved in ECP^32–41^ internalization.

Previous studies have emphasized that the use of CPPs should improve drug delivery to cells, although CPPs usually target cells promiscuously [46]. Most CPPs have high internalization rates *in vitro* but low target specificity *in vivo* [47]. Certain peptides, denoted cell-targeting peptides, specifically target a certain type(s) of cell(s) and bind to their target(s) strongly [48]. CPP fusion with cell-targeting peptide might therefore, prove useful as drug delivery systems, although however, TAT linked to antibody did not retain the cell-targeting ability of the antibody [46]. Nevertheless, Kuniyasu and colleagues, using phage display technology, isolated the peptide, CAYHRLRRC that contained a lymph node-homing sequence (CAY) and a cell-penetrating motif (RLRR) [49], which selectively penetrated leukaemia and lymphoma cells *in vitro*. Notably, we found that ECP^32–41^ could penetrate cells *in vitro* and selectively penetrate broncho-epithelial and intestinal villi tissues *in vivo* (Figure 7). ECP^32–41^ targets specific cells and tissues effectively and thus may be used in the development of innovative biomaterials for molecular detection and diagnosis purposes.

Most known CPPs are non-human in origin, which means that the adaptive immune response to these molecules will be of particular concern during the development of biomedical applications, especially if the CPP is conjugated to a protein or nanoparticle. The overall sequence identity among 13 primate eosinophilic RNases, each containing approximately 130 amino acids, is 67%. The sequence identities for ECP^32–41^ and the correspondent regions of human RNase 2 and RNase 8 are 80% and 50%, respectively, and those for other human RNases are smaller (Table S1). Interestingly, the corresponding 10-amino acid sequences of eosinophil RNases from higher primates, *Pan troglodytes* and *Gorilla gorilla*, the closest living relatives to humans [50], are identical to that of ECP^32–41^. In addition, the corresponding sequences in *Macaca fascicularis* and *Macaca nemestrina* RNases are 80% identical to ECP^32–41^ but are completely identical to that of EDN^32–41^ (Table S2). Therefore, residues 32–41 in ECP may have evolved from those in EDN. Apparently, residues 32–41 are not conserved in the members of the human RNase A superfamily, but represent a specific motif present in higher primates.

In summary, ECP^32–41^ is not cytotoxic and can be covalently coupled to many different molecules, it has a substantial cargo delivery potential as an attractive candidate for intracellular delivery of therapeutic molecules. GAG-mediated internalization may be the major pathway for ECP^32–41^ internalization. Finally, ECP^32–41^ is a human-derived peptide and specifically targets certain tissues, we expect that, with or without modification**,** it can be useful as a drug delivery system.

## Methods

### Peptide Design and Synthesis

Peptides with or without an *N*-terminally conjugated fluorescein isothiocyanate (FITC) group (Table 1) were synthesised by Genemed Synthesis Inc. and their purities (>90%) were assessed by analytical high-performance liquid chromatography. FITC was conjugated to *N*-terminus of ECP^32–41^ through a 5-carbon linker, which gave a spacer of approximately 10 angstroms in length. Peptide sequences were confirmed by matrix-assisted laser desorption/ionisation time-of-flight mass spectrometry in Genemed Synthesis Inc.

### Cell Cultures

Beas-2B cells were cultured in RPMI 1640 medium, 10% (v/v) heat-inactivated fetal bovine serum (FBS), 1% (v/v) Glutamine-Penicillin-Streptomycin (Biosera). Chinese hamster ovary (CHO) and AGS cells were maintained in Ham's F-12, 10% heat-inactivated FBS, 1% (v/v) Glutamine-Penicillin-Streptomycin. A549 cells were cultured in Dulbecco’s Modified Eagle Medium, 10% heat-inactivated FBS, 1% (v/v) Glutamine-Penicillin-Streptomycin. Caco-2 cells were grown in minimum essential medium, 10% FBS, 1% non-essential amino acids, 1% l-glutamine (Biowhittaker), 1% (v/v) Glutamine-Penicillin-Streptomycin. All cells were grown in a 5% CO_2_, humidified atmosphere at 37°C. Cell culture media, non-essential amino acids, and FBS were purchased from Invitrogen. Beas-2B, CHO, AGS, A549 and Caco-2 cells were purchased from ATCC.

### Cell-based ELISA

Cells (2×10^4^/well) were seeded into 96-well black plate and incubated under a 5% CO_2_ atmosphere at 37°C for 24 h. Each well was then washed with 150 µl ice-cold PBS. To prevent non-specific antibody binding, BSA was used as a blocking agent, and PBS containing 2% (w/v) BSA was added to each well at 4°C and incubated for 1 h. The wells were then washed with 100 µl ice-cold PBS. FITC-conjugated peptides were diluted to 10, 20, or 100 µM in PBS and then, medium (95 µl) and a peptide/PBS solution (5 µl) were gently mixed, added into a well, and the plate was placed on ice for 1 h. Each well was then washed with 100 µl PBS and the FITC fluorescent intensity for each sample was measured using a fluorescence spectrophotometer (Wallac Victor II, Perkin Elmer, USA) and excitation and emission wavelengths of 485 nm and 521 nm respectively.

### Flow Cytometry

Cells (3.0×10^5^/well) were added into six-well plates and cultured in the indicated medium. After 24 h, each FITC-peptide, dissolved in medium, was added into a well and the samples incubated for 1 h. Cells were then harvested, washed, and suspended in PBS. The fluorescent intensities of the cell samples were measured using a FACSCalibur flow cytometer (BD Biosciences, Franklin Lakes, NJ) and excitation and emission wavelengths of 488 nm and 515–545 nm respectively. The relative internalization of each peptide is reported as the mean ﬂuorescent signal for 10,000 cells.

### Confocal Laser-scanning Microscopy (CLSM)

Cells were cultured on coverslips (1.0×10^4^/coverslip) in indicated medium. After 24 h, cell samples were each incubated at 37°C for 1 h with an FITC-peptide. The cells were then washed twice with PBS, fixed with 2% (w/v) paraformaldehyde and incubated, first in PBS for 15 min, then with 50 mM NH_4_Cl in PBS for 10 min, and finally permeabilised with 0.5% (v/v) Triton-X-100 at 25°C for 5 min. Nuclei were stained with Hoechst 33342 Fluorescent Stain, **(**Sigma-Aldrich**)** during the final 5 min of incubation. Cells were then washed twice with 0.05% Triton-X-100, once with PBS, and the coverslips were mounted in a Vectashield anti-fade mounting medium (Vector Labs). CLSM was performed using LSM510 META (Carl Zeiss, Göttingen, Germany) to assess the distribution of the FITC-peptides in the cells.

### GAG Competition Assay

Beas-2B cells (2×10^4^/well) in RPMI 1640 medium were seeded into 96-well black plate for cell binding test, while cells (3.0×10^5^/well) incubated in the wells of six-well plates were used for internalization test. After 24 h, cells were treated for 30 min with 0.01, 0.1, 1, 5, 10, 50, or 100 µg/ml of low molecular weight heparin (LMWH; average M_r_ ∼3,000), chondroitin sulfate C (CSC; average M_r_ ∼50,000–58,000), or HA (M_r_ ∼3,000,000–5,800,000), all obtained from Sigma-Aldrich (St. Louis, MO, USA). The cells were then incubated with FITC-ECP^32–41^ and assayed for cell binding or internalization of ECP^32–41^using cell-based ELISA or flow cytometry, respectively, as described above.

### Heparinase and Chondroitinase ABC Depletion of GAGs

Beas-2B cells (3.0×10^5^/well) were incubated with RPMI 1640 medium overnight in six-well plates and then treated with 5 U/ml of heparinase I, 2.4 U/ml of heparinase III, or 10 U/ml of chondroitinase ABC (Sigma-Aldrich) at 37°C for 2 h. After a PBS wash, the cells were incubated with FITC-ECP^32–41^ and assayed for internalization of the peptide by flow cytometry as described above.

### Cell Internalization Pathway

The influence of energy on ECP^32–41^ internalization was measured at 4°C, 37°C, and while depleting ATP with 10 mM sodium azide and 6 mM 2-deoxy-d-glucose (Sigma-Aldrich) at 37°C for 30 min. Beas-2B cells were treated with 10 µM chlorpromazine, 5 mM methyl-β-cyclodextrin, 25 µM genistein, 2 µM filipin III, 4 µM cytochalasin D, 20 µM nocodazole, 50 nM wortmannin or 50 µM dimethyl amiloride at 37°C for 30 min (Sigma-Aldrich). After treatment, the cells were incubated with FITC-ECP^32–41^ and assayed by flow cytometry as described above.

### Cell Viability Assay

The effects of the peptides on cell viability were determined colourimetrically using 3-(4,5-dimethylthiazol-2-yl)-2,5-diphenyltetrazolium bromide (MTT) (US Biological). Cells (1.0×10^4^/well) were seeded into the wells of 96-well plates and incubated overnight. Cell samples were then exposed to different concentrations of TAT^47–57^, ECP^32–41^, KLA, KLA-TAT^47–57^ or KLA-ECP^32–41^. After 24 h, 100 µl of 0.5 mg/ml MTT in medium was added into each well, and the cells were incubated at 37°C for 3 h. The incubation medium was removed, and the remaining purple crystal formazan was dissolved in dimethylsulphoxide. Cells treated with 0.1% Triton X-100 was used as a positive control for cell viability. A_540_ values were measured using a multiwall plate reader (Molecular Devices).

### Membrane Disruption Assay

Lactate dehydrogenase (LDH) was used to quantify membrane disruption. The release of LDH from cells was measured by Promega CytoTox-ONE assay (Promega, USA). Cells (1.0×10^4^/well) were seeded into the wells of 96-well plates and incubated overnight. Cell samples were then exposed to different concentrations of ECP^32–41^. After 24 h, 100 µl of extracellular medium was transferred to a black 96-well plate containing 100 µl of CytoTox-ONE reagent, incubated at RT for 10 min. Fluorescent intensity for each sample was measured using a fluorescence spectrophotometer (Wallac Victor II, Perkin Elmer, USA) and excitation and emission wavelengths of 540 nm and 590 nm respectively. LDH released from cells lysed with 0.1% Triton X-100 in medium was defined as 100% leakage and LDH released from untreated cells was set as 0% leakage.

### Subcellular Fractionation

Beas-2B cells (8×10^5^/dish) were cultured in 10 cm dish for 24 h, followed by incubation with 20 µM eGFP or eGFP-ECP^32–41^ at 4°C for 1 h. The cells were washed twice with PBS and then incubated at 37°C for 1 h, 2 h, 3 h and 4 h, separately. Cells were then homogenized and fractionated by floatation in Percoll gradients (GE Healthcare, USA) separating cytoplasm and endosomes [51]. In brief, cells were scraped off in 1 ml PBS with a rubber policeman and pelleted at 300×*g* for 5 min. The pellet was resuspended in 1 ml homogenization buffer (0.25 M sucrose, 3 mM Imidazole and 0.5 mM EDTA, pH 7.3) and pelleted again at 800×*g* for 7 min. The pellet was resuspended in 100 µl homogenization buffer with a syringe until the cells were broken but the nuclei were still intact as observed by light microscopy. The homogenate was diluted to a total volume of 1 ml with homogenization buffer. After homogenization, the gold-filled fraction was pelleted together with the nuclei at 800×*g* for 7 min. The pellet was resuspended in 650 µl 17% Percoll and loaded onto a 500 µl 64% sucrose cushion in a 2 ml Beckman ultracentrifuge tube. The samples were centrifuged for 90 min at 27,000×*g* in a Beckman SW55Ti rotor with fast acceleration to distribute the nuclear fraction at the top and the endosome-filled organelles at the bottom of the sucrose cushion. The pellet was resuspended in 100 µl homogenization buffer and referred to endosomal fraction in the results.

### Western Blotting

Protein concentration from each fraction was estimated by BCA protein assay kit (Thermo). Proteins were resolved as reported in 12% SDS-PAGE and blotted to BioTrace™ polyvinylidene fluoride Membrane (Pall Life Sciences, USA). The membrane was incubated in blocking solution (5% nonfat dry milk in PBS) for 1 h. Blots were incubated with antibodies for anti-actin (Novus Biologicals, CO), anti-lysosomal-associated membrane protein 1 (LAMP-1) (Santa Cruz Biotechnology, CA) and anti-His (Clontech Laboratories, CA) in PBS with 0.1% Tween 20 (TPBS) for 1 h. After wash with TPBS for 10 min three times, the membrane was incubated with horseradish peroxidase-conjugated anti-mouse IgG in TPBS at 25°C for 1 h. After wash with TPBS for 10 min three times, the protein on membrane was detected using chemiluminescent detection kit (ECL, Amersham Life Science) and chemiluminescence was measured by Kodak X-Omat film. The blotted signal was quantitated using NIH ImageJ software.

### Immunohistochemical Staining

Adult female specific-pathogen-free Sprague-Dawley rats (Narl:SD) with body weights between 200 and 300****g were purchased from, and maintained at, the National Laboratory Animal Center, Taiwan. The rats were separated into two groups and injected with 5 nmol of enhanced green fluorescence protein (eGFP) or eGFP-ECP^32–41^ through their tail veins. All animals were asphyxiated with CO_2_, 1 h after injection. All major organs including brain, heart, lung, trachea, kidney, liver, spleen and intestine were removed and immediately fixed in 10% neutral-buffered formaldehyde. The tissue samples were processed by standard methods to prepare paraffin wax-embedded block samples [25]. The blocks were sectioned into 6 µm slices and were examined using a Super Sensitive Non-Biotin HRP Detection System (BioGenex Laboratories, San Ramon, CA) as previously described [25]. All these slices were then observed by using light microscope (Zeiss-Axioplan, Germany).

### Statistical Analysis

Each result is reported as the mean ± standard deviation (SD), where *n* is the number of experiments. To compare two means, statistical analysis was performed using the unpaired Student’s *t*-test in GraphPad Prism v4.02 (GraphPad Software, USA). One-way analysis of variance (ANOVA), followed by Dunnett’s test, was used to test for differences among multiple treatments. A *P* value <0.05 was considered to be statistically significant.

## Supporting Information

Figure S1 **eGFP-ECP^32–41^ in endosomal fraction.** (A) Beas-2B cells were incubated with eGFP or eGFP-ECP^32–41^ at 4°C for 1 h. The cells were washed twice with PBS and then shifted to 37°C for further 1 h, 2 h, 3 h or 4 h. Cells were then homogenized and fractionated by floatation in Percoll gradients separating cytoplasm and endosomes. The locations of eGFP or eGFP-ECP^32–41^ were analysed by Western blot. (B) The blotted signal was quantitated using NIH ImageJ software and normalized to LAMP-1. The internalization of cells treated with eGFP-ECP^32–41^ for 2 h was set to 100%. The result is expressed as the mean ± S.D., *n* = 3. *, *P*<0.05.
(TIF)

Figure S2 **Cell-surface GAG-dependent cytotoxicity of KLA-ECP^32–41^.** GAG-mediated inhibition of KLA-ECP^32–41^ peptide-induced cytotoxicity in Beas-2B cells. Beas-2B cells were treated with increasing concentrations of LMWH, CSC or HA for 30 min prior to addition of 10 *µ*M KLA-ECP^32–41^ at 37°C for 24 h. The cytotoxicity of KLA-ECP^32–41^ was determined by an MTT assay. The cell viability untreated cells was set to 100%. The result is expressed as the mean ± S.D., *n* = 3.
(TIF)

Table S1 (DOC)

Table S2 (DOC)
