# Supplementary figures and images for: A Novel Cell-Penetrating Peptide Derived from Human Eosinophil Cationic Protein
Source: PLoS One. 2013 Mar 4;8(3):e57318. doi: 10.1371/journal.pone.0057318 (PMC3587609; doi:10.1371/journal.pone.0057318)

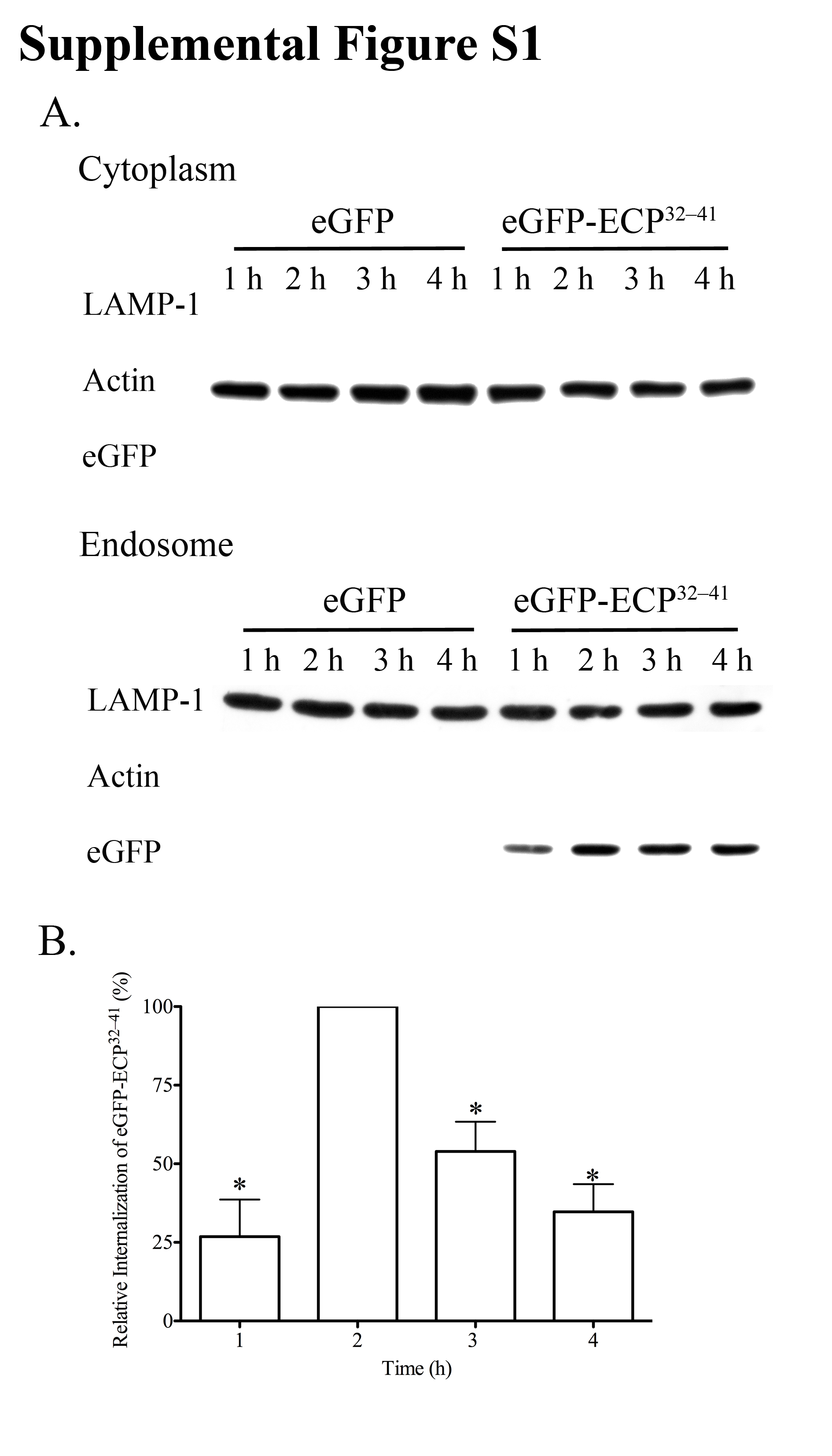

Supplement: Figure S1 — eGFP-ECP32–41 in endosomal fraction. (A) Beas-2B cells were incubated with eGFP or eGFP-ECP32–41 at 4°C for 1 h. The cells were washed twice with PBS and then shifted to 37°C for further 1 h, 2 h, 3 h or 4 h. Cells were then homogenized and fractionated by floatation in Percoll gradients separating cytoplasm and endosomes. The locations of eGFP or eGFP-ECP32–41 were analysed by Western blot. (B) The blotted signal was quantitated using NIH ImageJ software and normalized to LAMP-1. The internalization of cells treated with eGFP-ECP32–41 for 2 h was set to 100%. The result is expressed as the mean ± S.D., n = 3. *, P<0.05. (TIF) [file pone.0057318.s001.tif]

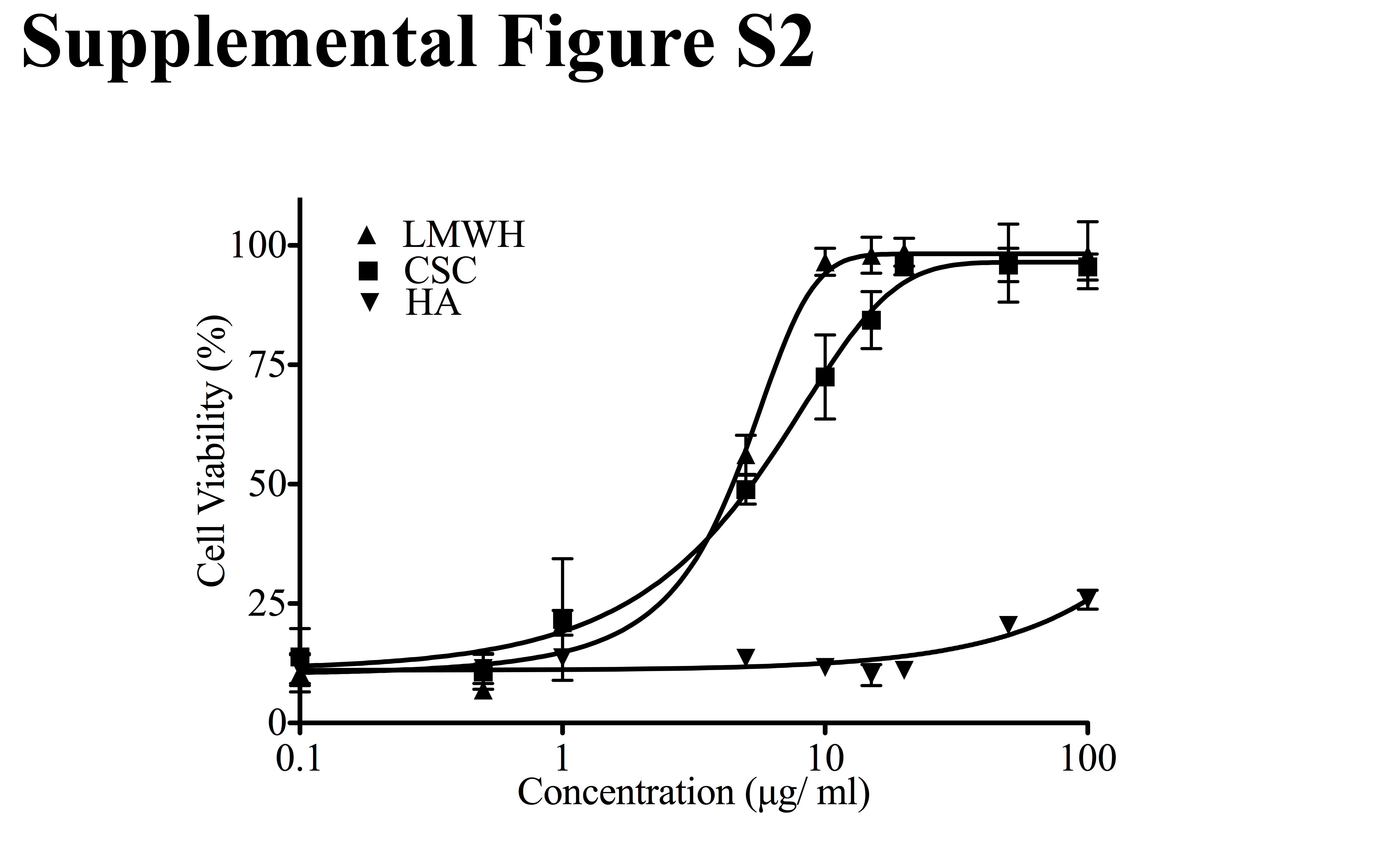

Supplement: Figure S2 — Cell-surface GAG-dependent cytotoxicity of KLA-ECP32–41. GAG-mediated inhibition of KLA-ECP32–41 peptide-induced cytotoxicity in Beas-2B cells. Beas-2B cells were treated with increasing concentrations of LMWH, CSC or HA for 30 min prior to addition of 10 µM KLA-ECP32–41 at 37°C for 24 h. The cytotoxicity of KLA-ECP32–41 was determined by an MTT assay. The cell viability untreated cells was set to 100%. The result is expressed as the mean ± S.D., n = 3. (TIF) [file pone.0057318.s002.tif]
