# Supplementary material for: A Novel Cell-Penetrating Peptide Derived from Human Eosinophil Cationic Protein
Source: PLoS One. 2013 Mar 4;8(3):e57318. doi: 10.1371/journal.pone.0057318 (PMC3587609; doi:10.1371/journal.pone.0057318)
Supplement: Table S1 — (DOC) [file pone.0057318.s003.doc]

**Supporting Information**

**Supplemental Table S1**

**Comparison of ^32^NYRWRCKNQN^41^ motif among human RNase A members**

| **Name** | **Protein identity** | **Motif sequence** | **ECP^32–41^ identity** |
| --- | --- | --- | --- |
| RNase1 | 30% | MTQGRCKPVN | 40% |
| RNase2 | 67% | NYQRRCKNQN | 80% |
| RNase3 | 100% | NYRWRCKNQN | 100% |
| RNase4 | 28% | MTLYHCKRFN | 30% |
| RNase5 | 32% | LTSP-CKDIN | 30% |
| RNase6 | 43% | KYFGRSLELY | 20% |
| RNase7 | 39% | KHTKRCKDLN | 40% |
| RNase8 | 39% | KYTERCKDLN | 50% |
| RNase9 | 23% | YYKHRWVAEH | 20% |
| RNase10 | 26% | EPSQSCIAQY | 10% |
| RNase11 | 30% | EANGSCKWSN | 30% |
| RNase12 | 23% | EPDHTCKKEH | 20% |
| RNase13 | 25% | MQNSDCPKIH | 10% |

Sequence identity was performed with National Center for Biotechnology Information Blast (*NCBI Blast:* <http://blast.ncbi.nlm.nih.gov/Blast.cgi>).
