# Supplementary material for: A Novel Cell-Penetrating Peptide Derived from Human Eosinophil Cationic Protein
Source: PLoS One. 2013 Mar 4;8(3):e57318. doi: 10.1371/journal.pone.0057318 (PMC3587609; doi:10.1371/journal.pone.0057318)
Supplement: Table S2 — (DOC) [file pone.0057318.s004.doc]

**Supporting Information**

**Supplemental Table S2**

**Comparison of ^32^NYRWRCKNQN^41^ motif among primate ECPs and EDNs**

| **Protein** | **Organism** | **Protein identity** | **Motif**  **Sequence** | **ECP^32–41^ identity** |
| --- | --- | --- | --- | --- |
| **ECP** | *Homo sapiens* | 100% | NYRWRCKNQN | 100% |
|  | *Pan troglodytes* | 97% | NYRWRCKNQN | 100% |
|  | *Gorilla gorilla* | 97% | NYRWRCKNQN | 100% |
|  | *Macaca fascicularis* | 88% | NYQRRCKNQN | 80% |
|  | *Macaca nemestrina* | 88% | NYQRRCKNQN | 80% |
|  | *Pongo pygmaeus* | 88% | NYQRRCKDQN | 70% |
| **EDN** | *Homo sapiens* | 67% | NYQRQCKNQN | 80% |
|  | *Pan troglodytes* | 67% | NYQRQCKNQN | 80% |
|  | *Gorilla gorilla* | 69% | NYQRQCKNQN | 80% |
|  | *Macaca fascicularis* | 67% | NYQRQCKNQN | 80% |
|  | *Macaca nemestrina* | 66% | NYQRQCKNQN | 80% |
|  | *Pongo pygmaeus* | 68% | NFQRRCKNQN | 70% |

Sequence identity was performed with National Center for Biotechnology Information Blast (*NCBI Blast:* <http://blast.ncbi.nlm.nih.gov/Blast.cgi>).
